# Supplementary material for: Pandemic-Potential Viruses are a Blind Spot for Frontier Open-Source LLMs
Source: medRxiv. 2025 Dec 5:2025.12.04.25341642. Preprint. [Version 1] doi: 10.64898/2025.12.04.25341642 (PMC12706609; doi:10.64898/2025.12.04.25341642)
Supplement: 1 [file NIHPP2025.12.04.25341642V1-supplement-1.pdf]

medRxiv preprint doi: https://doi.org/10.64898/2025.12.04.25341642; this version posted December 5, 2025. The copyright holder for this preprint (which was not certified by peer review) is the author/funder, who has granted medRxiv a license to display the preprint in perpetuity.

It is made available under a CC-BY 4.0 International license .

A Appendix

A.1 Additional Methods

Common-sense filters for data preprocessing Patient age was restricted to non-negative values, systolic blood pressure (SBP) to ≤200 mmHg, diastolic blood pressure (DBP) to ≤150 mmHg, pulse rate to ≤200 beats per minute, respiratory rate to ≤60 breaths per minute, and body weight to ≤300 kg. In addition, encounter dates were required to be valid calendar entries from the year 2020 onward. Records failing these criteria were excluded (records with missing values were retained).

Generation of training and testing splits To generate training and testing datasets, we first excluded samples with neither a *viral* nor a *non-viral* ground truth label and retained only those with confirmed positive or negative outcomes. The binary outcome labels were extracted as the dependent variable (y), while all remaining structured clinical and epidemiological features (excluding outcome labels) were used as predictors (X). We then split the data into training (80%) and testing (20%) sets using a stratified train–test split to preserve class balance, with a fixed random seed (42) to ensure reproducibility.

Random forest model Numerical and categorical variables were identified based on curated data labels. Numerical variables were standardized using `StandardScaler`, while categorical variables were one-hot encoded with `OneHotEncoder` (ignoring unseen categories at test time). Preprocessing steps were implemented in a `ColumnTransformer` pipeline to ensure consistent transformations between training and testing data. Irrelevant features, such as patient identifiers, were excluded.

The model was trained on the training set using `RandomForestClassifier` with 100 trees (`n_estimators`=100). Both training and evaluation were repeated across three different random seeds (1, 42, 120) to ensure reproducibility. We also trained models on randomly permuted outcome labels (*scrambled* condition), which consistently yielded performance at random baseline (Figure S2).

Feature importance scores were extracted from trained forests. To aid interpretation, we also derived reduced feature sets (top 20, 10, and 5 features by importance determined by the random forest model trained on all features and features most frequently available in hospital settings) and retrained the random forest model on each set (Figure S2).

XGBoost model We implemented a gradient boosting baseline using `XGBoostClassifier`. Numerical and categorical variables were standardized and one-hot encoded using the same `ColumnTransformer` pipeline to ensure identical transformations between training and testing. Irrelevant identifiers (e.g., patient identifiers) were excluded prior to model fitting.

The XGBoost model was trained on the training split with 600 estimators (`n_estimators=600`), maximum tree depth of 5 (`max_depth=5`), learning rate of 0.05, and regularization parameters `reg_lambda=1.0` and `min_child_weight=1.0`. Subsampling and column sampling ratios were set to 0.8 (`subsample` and `colsample_bytree`) to mitigate overfitting. Models were trained with the `binary:logistic` objective and `AUC` evaluation metric. Early stopping was applied with a patience of 50 rounds based on validation AUC, and training was repeated across three random seeds (1, 42, 120) for reproducibility. GPU acceleration (`tree_method="gpu_hist"`) was enabled.

To confirm that observed performance was not driven by dataset artifacts, we also trained models on randomly permuted outcome labels (*scrambled* condition), which consistently produced AUC values at random baseline.

Medical Context Summaries Records from 20 *viral* and 20 *non-viral* patients were randomly chosen from the training data, converted to JSON, and grouped into batches that fit within the LLM context window. Each batch was submitted to a local LLM (`gpt-oss-120b`, temperature=0.3) together with the structured field definitions. The model was instructed to extract only high-value patterns and return a concise set of rules. Summaries were returned as bullet-point lists grouped into *viral indicators* and *non-viral indicators* (plus *RF interpretation rules* when RF predictions were included). When RF predictions were included, the batch prompt moreover included a list of the most important features identified by the RF model.

medRxiv preprint doi: https://doi.org/10.64898/2025.12.04.25341642; this version posted December 5, 2025. The copyright holder for this preprint (which was not certified by peer review) is the author/funder, who has granted medRxiv a license to display the preprint in perpetuity.

It is made available under a CC-BY 4.0 International license .

In a second stage, all batch summaries were merged with another LLM call, which deduplicated overlapping items and produced a single compact knowledge base. This distilled summary was later provided to downstream models as the medical context. The exact batch and merging prompts are provided in Box A.2 and Box A.3.

medRxiv preprint doi: https://doi.org/10.64898/2025.12.04.25341642; this version posted December 5, 2025. The copyright holder for this preprint (which was not certified by peer review) is the author/funder, who has granted medRxiv a license to display the preprint in perpetuity.

It is made available under a CC-BY 4.0 International license .

Table A1: User prompts used in different context settings.

**Context Setting**	**User Prompt**
Zero-shot	`Patient data:`<br>`{patient_text}`
Medical context	`Use the distilled knowledge base to guide your prediction.`<br><br>`Knowledge base:`<br>`{knowledge_summary}`<br><br>`Now, analyze the current patient:`<br>`{patient_text}`
RAG	`Viral diagnoses (1=pos, 0=neg) of the top {top_k} most similar patients:`<br>`{retrieved_block}`<br><br>`Now, analyze the current patient:`<br>`{patient_text}`

medRxiv preprint doi: https://doi.org/10.64898/2025.12.04.25341642; this version posted December 5, 2025. The copyright holder for this preprint (which was not certified by peer review) is the author/funder, who has granted medRxiv a license to display the preprint in perpetuity.

It is made available under a CC-BY 4.0 International license .

Box A.1: LLM System Prompt

```
You are an expert infectious disease physician and public health
expert,
helping to prioritize patients for viral pathogen detection based on
their clinical data.

The patient metadata will be structured as a JSON object with some of
the following fields:
{field_definitions}
Use this additional information about the fields to inform your
predictions.

From the metadata, decide:
1. Is the case viral? (yes/no)
2. Probability of viral (%)

Respond only in this exact format:
Viral: <yes / no>
Probability of viral: <percentage>

Base your answer only on the data. If unsure, output unknown.
```

medRxiv preprint doi: https://doi.org/10.64898/2025.12.04.25341642; this version posted December 5, 2025. The copyright holder for this preprint (which was not certified by peer review) is the author/funder, who has granted medRxiv a license to display the preprint in perpetuity.

It is made available under a CC-BY 4.0 International license .

Box A.2: LLM Medical Context Batch Prompt (with RF)

```
You are an expert clinical reasoning engine analyzing a dataset of
labeled patient cases.

Each case includes:
- Structured patient metadata (symptoms, demographics, exposures,
comorbidities)
- A ground truth diagnosis ('viral_diagnosis')
- Predictions from a random forest model ('probability_of_viral_rf'
and 'viral_rf')

The RF model found the following features to be highly predictive.
Critically assess their clinical plausibility:
{feature_importance_text}

Your task:
Distill only the most essential, high-value insights that will help
another model predict viral vs.  non-viral cases.  Keep your summary
short.  Focus on what is consistently important and ignore less
relevant patterns.

Specifically:
- Key symptom/exposure patterns strongly linked to viral infection.
- Key patterns strongly linked to non-viral cases.
- How RF predictions align or misalign with true outcomes.
- When to trust or override the RF outputs.

Output format:
A concise bullet-point list grouped into:
1.  "Viral indicators"
2.  "Non-viral indicators"
3.  "RF interpretation rules"

Do not include explanations, background context, or verbose narrative.
Only include the minimal set of rules and patterns essential for
training the next model.

Here is the training data:
```

medRxiv preprint doi: https://doi.org/10.64898/2025.12.04.25341642; this version posted December 5, 2025. The copyright holder for this preprint (which was not certified by peer review) is the author/funder, who has granted medRxiv a license to display the preprint in perpetuity.

It is made available under a CC-BY 4.0 International license .

Box A.3: LLM Medical Context Merging Prompt (with RF)

```
You are an expert in infectious diseases and clinical data analysis.

Your task:
Merge the following batch summaries into one concise, unified
clinical knowledge base that will help another model make accurate
viral vs.  non-viral predictions.

Include only:
- Consistent, high-value relationships between exposures, symptoms,
and pathogens.
- Key patterns that strongly indicate viral or non-viral status.
- Notable exceptions and rare but important edge cases.
- Key takeaways from Random Forest predictions:  when to trust or
override them, and the most important features.

Output format:
A short bullet-point list grouped into:
1.  "Viral indicators"
2.  "Non-viral indicators"
3.  "RF interpretation rules"

Instructions:
- Eliminate repetition from the batch summaries.
- Ignore low-value or inconsistent observations.
- Do not include background explanations or long narratives.
- Focus only on rules and patterns essential for model training.

Below are the batch summaries:
```

medRxiv preprint doi: https://doi.org/10.64898/2025.12.04.25341642; this version posted December 5, 2025. The copyright holder for this preprint (which was not certified by peer review) is the author/funder, who has granted medRxiv a license to display the preprint in perpetuity.

It is made available under a CC-BY 4.0 International license .

B Supplementary Figures

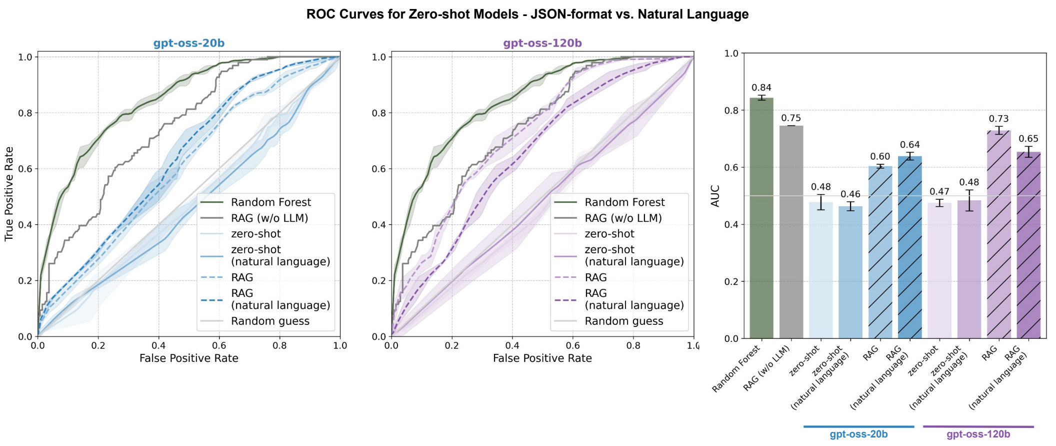

Figure S1: **Patient data in structured JSON format vs. natural language.** To rule out the possibility that model performance was limited by difficulties in interpreting patient data in structured JSON format, we also evaluated the LLMs using prompts with the patient data converted to natural language. This modification did not affect the performance of the model.

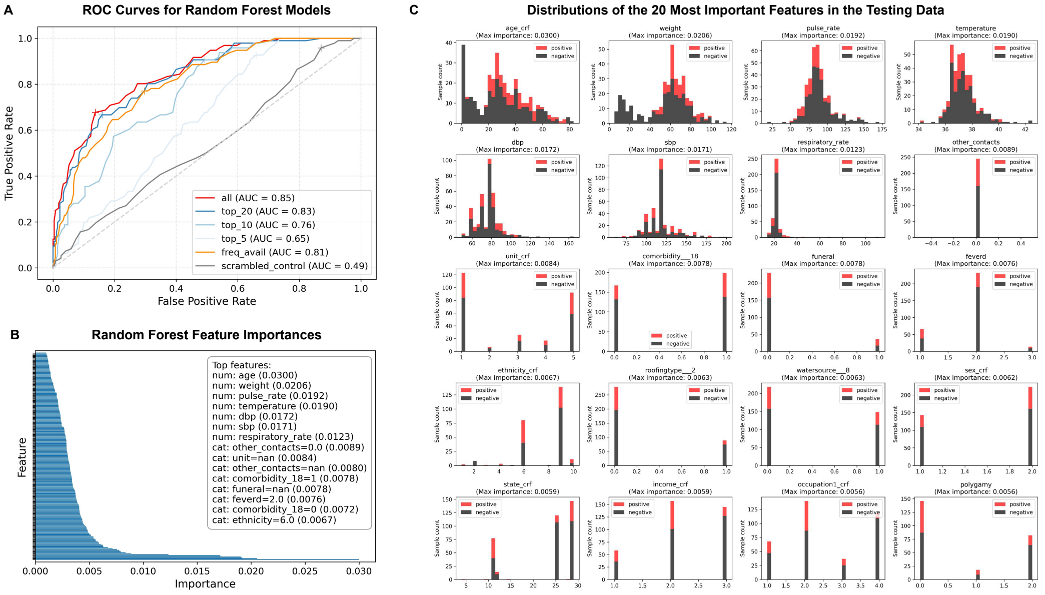

Figure S2: **Performance and feature analysis of random forest models. (A)** Receiver operating characteristic (ROC) curves for RF models trained on different subsets of clinical features: all features, the top 20, top 10, and top 5 as identified by the full RF model, as well as routinely collected clinical features (*freq_avail*). **(B)** Ranked feature importances of the RF model trained on all features, highlighting the strongest numerical (*num*) and categorical (*cat*) predictors. **(C)** Distributions of the 20 most important features identified by the RF model trained on all features in the testing data, stratified by ground-truth infection status: *viral* (positive) and *non-viral* (negative).

medRxiv preprint doi: https://doi.org/10.64898/2025.12.04.25341642; this version posted December 5, 2025. The copyright holder for this preprint (which was not certified by peer review) is the author/funder, who has granted medRxiv a license to display the preprint in perpetuity.

It is made available under a CC-BY 4.0 International license .

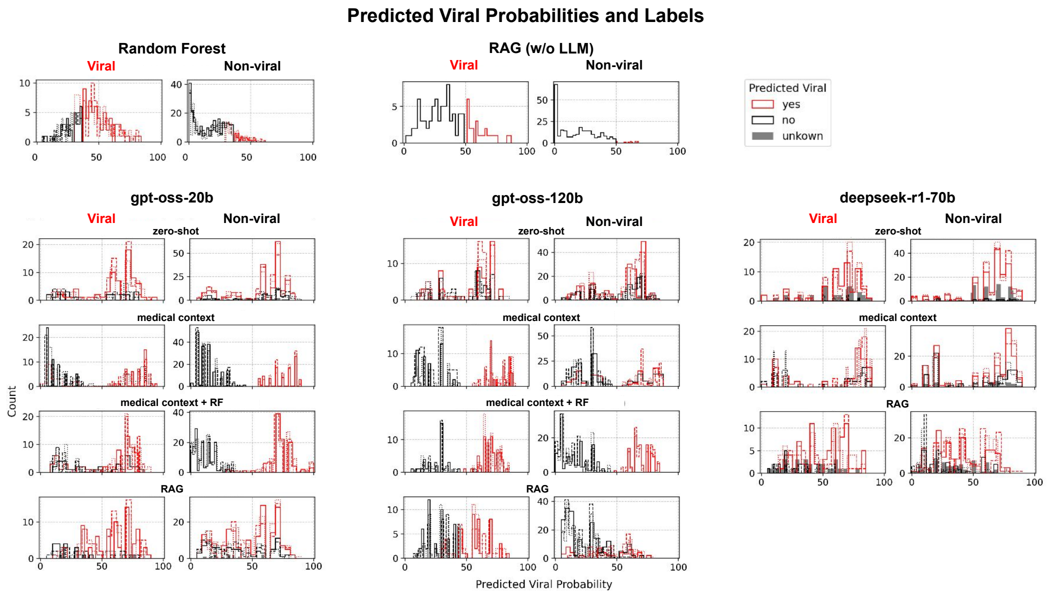

Figure S3: **Distribution of predicted viral probabilities and binary labels across models and context settings.** Each panel shows histograms of predicted probabilities stratified by ground-truth status: *viral* (left column) and *non-viral* (right column). Rows correspond to different models and context settings. The different line styles (solid, dashed, and dash–dot) correspond to three independent LLM inference runs (temperature = 0.5) or random seeds for the RF model.

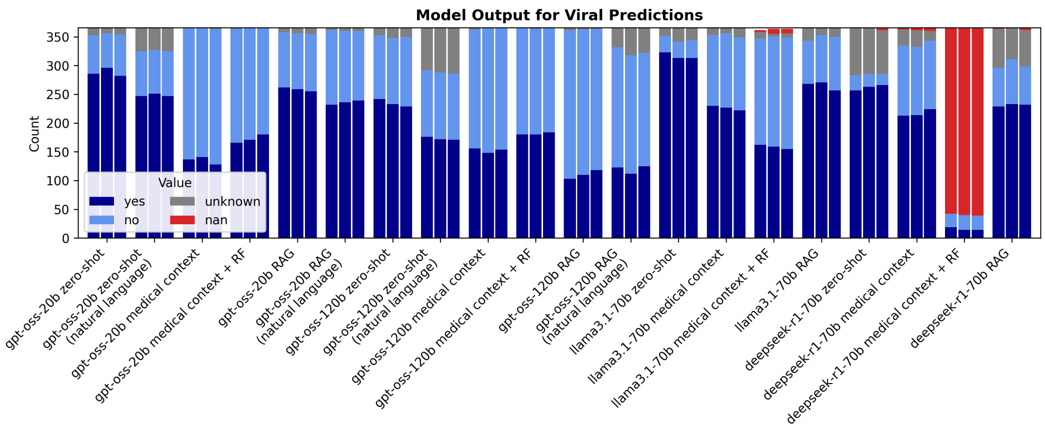

Figure S4: **Model outputs for viral predictions across different LLMs and context settings.** Bars indicate the occurrence of predicted labels ("yes", "no", "unknown", or missing ("nan")) for each model–context combination. While models were explicitly prompted to return "unknown" when insufficient data were available, this response was infrequent, with most outputs falling into binary yes/no categories. The *deepseek-r1-70b medical context + RF* configuration produced large fractions of missing values due to context window limitations deriving from the computational resources that we had available for this study, and this configuration was therefore omitted from the rest of this study.

medRxiv preprint doi: https://doi.org/10.64898/2025.12.04.25341642; this version posted December 5, 2025. The copyright holder for this preprint (which was not certified by peer review) is the author/funder, who has granted medRxiv a license to display the preprint in perpetuity.

It is made available under a CC-BY 4.0 International license .

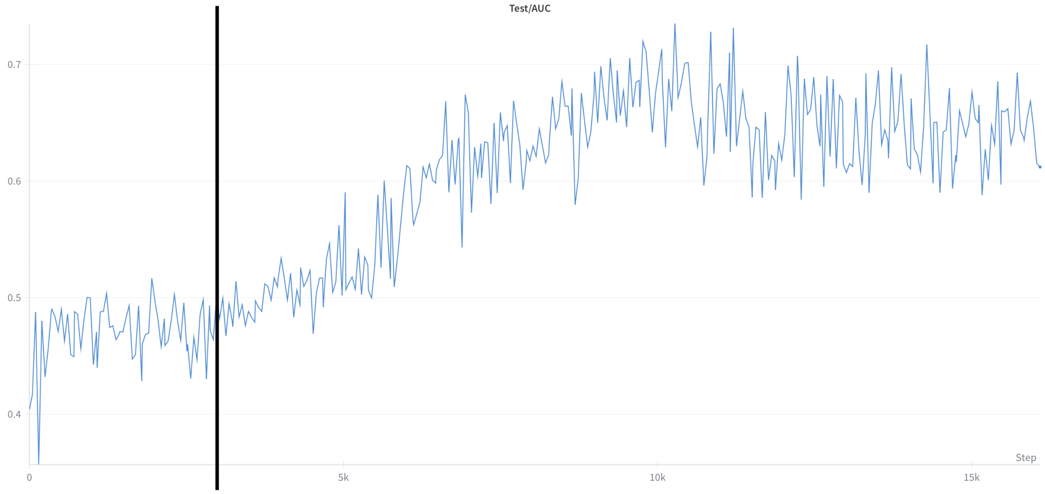

Figure S5: **Training dynamics of Gemma-4B-RL.** The model exhibits an extended plateau phase (black line) before entering a period of rapid improvement. Shown is the AUC on a balanced test set.
